# Multiple antiviral mechanisms of Ephedrae Herba and Cinnamomi Cortex against influenza: inhibition of entry and replication

**DOI:** 10.1128/spectrum.00371-25

**Published:** 2025-04-30

**Authors:** Aya Fujikane, Ryosuke Fujikane, Yusuke Sechi, Akinori Nishi, Yoshizumi Ishino, Tetsuya Hiyoshi, Atsuhiko Sakamoto, Shigeki Nabeshima

**Affiliations:** 1 Department of General Medicine, Faculty of Medicine, Fukuoka University 38068 , Fukuoka, Fukuoka Prefecture, Japan; 2 Department of Physiological Science and Molecular Biology, Fukuoka Dental College 12763, Fukuoka, Fukuoka Prefecture, Japan; 3 Oral Medicine Research Center, Fukuoka Dental College 12763, Fukuoka, Fukuoka Prefecture, Japan; 4 TSUMURA Advanced Technology Research Laboratories, TSUMURA & CO. 47740 , Inashiki-gun, Ibaraki Prefecture, Japan; 5 Department of Bioscience and Biotechnology, Graduate School of Bioresource and Bioenvironmental Sciences, Kyushu University 318519 , Fukuoka, Fukuoka Prefecture, Japan; University of Georgia, Athens, Georgia, USA

**Keywords:** influenza virus, cap-dependent endonuclease, haemagglutinin, maoto, Ephedrae Herba, Cinnamomi Cortex

## Abstract

Maoto, a traditional herbal medicine widely prescribed in Japan, has been shown to be effective in the treatment of influenza virus infection, but the mechanisms of its antiviral action remain unclear. We previously demonstrated that maoto binds to respiratory syncytial virus (RSV) spike proteins, thereby inhibiting their entry into host cells. In this report, a similar experiment was done to determine if maoto and its components have an anti-infective effect on the influenza virus. Our results indicate that maoto binds to the hemagglutinin (HA) spike protein, inhibiting virus entry into host cells in a manner analogous to its antiviral effect on RSV. This hemagglutinin-binding effect was observed across influenza A(H1N1), A(H3N2), and B viruses, highlighting the broad-spectrum inhibitory potential of maoto against diverse viral strains. Furthermore, maoto, internalized by cells along with the influenza virus, binds to a cap-dependent endonuclease (polymerase acidic [PA] protein) that is crucial for viral replication and inhibits its nuclease activity. Among maoto’s constituent crude drugs, Ephedrae Herba (EH) and Cinnamomi Cortex (CC) were found to bind to both hemagglutinin and PA, indicating that they are responsible for the anti-infective effect of maoto. Maoto is distinctive in its multiple points of antiviral action, exhibiting a broad spectrum of antiviral properties, which makes it a versatile therapeutic agent against various viral mutations.

**IMPORTANCE:**

The influenza virus is a formidable pathogen responsible for global pandemics that claim over 300,000 lives annually. Employing an ingenious evolutionary strategy, this virus undergoes constant mutation, deftly evading the action of therapeutic agents and sustaining its relentless impact. Maoto, a traditional herbal medicine, has long been known for its efficacy against viral infections and is frequently prescribed in Japan for the treatment of influenza; however, the precise mechanisms of its action remain unclear. Our study was done to elucidate the antiviral mechanisms of maoto against the influenza virus, presenting data that supports its unique potential as a therapeutic agent capable of flexibly adapting to mutations of the influenza virus. These findings pave the way for the development of new drugs and the expansion of therapeutic options.

## INTRODUCTION

Influenza, which is caused by infection with the influenza A or B virus, has long posed a significant health threat. The coronavirus disease 2019 pandemic temporarily suppressed the global spread of seasonal influenza, but recent data indicate a resurgence (1). Despite advances over the past few decades, including the development of potent antiviral drugs and vaccines, we do not yet have the ability to fully suppress influenza epidemics and related fatalities. Currently, two classes of antivirals are FDA-approved for influenza A and B treatment: neuraminidase inhibitors (NAI) and cap-dependent endonuclease inhibitors (CENI) (2). The efficacy of these treatments is greatly influenced by host risk factors, the emergence of drug-resistant strains, genetic mutations, and reassortment of viral gene segments. Additionally, the potential for highly pathogenic avian influenza strains, such as H5 and H7, to evolve into forms capable of human-to-human transmission remains a serious concern. To better control influenza, it is crucial to develop new antiviral drugs and treatment strategies that operate through novel mechanisms, complementing existing NAI and CENI therapies.

Traditional herbal medicines (THMs) have long played an important role in public healthcare across the Far East, particularly in Japan, China, and Korea. The national medical insurance system of Japan has recently approved THMs, enabling physicians to prescribe these treatments more widely (3). Maoto, a traditional herbal medicine known as Ma-Huang-Tang in Chinese, has long been prescribed to treat acute febrile illnesses such as influenza, although its precise antiviral mechanisms remain unclear. Previous clinical trials (4–7) and experimental murine infection studies (8–11) have demonstrated maoto’s effectiveness against influenza. One of its known antiviral mechanisms is the ability to block the viral uncoating process by inhibiting the acidification of endosomes, thereby trapping the virus within these compartments (9). Maoto is a multi-component formulation derived from four plants: Ephedrae Herba (EH), Cinnamomi Cortex (CC), Armeniacae Semen (AS), and Glycyrrhizae Radix (GR). Of these, only EH and CC exhibit antiviral properties (12). In addition to its antiviral action, maoto possesses antitussive and antipyretic effects, making it a promising treatment for influenza.

We previously investigated the efficacy of maoto on respiratory syncytial virus (RSV) infection, finding that it, as well as its components EH and CC, exhibited antiviral effects in both *in vitro* and *in vivo* experiments (12, 13). RSV contains two outer spike proteins on its envelope: G, which is responsible for receptor adhesion, and F, which mediates membrane fusion (14, 15). Our research demonstrated that extracts from EH and CC interact with the central conserved domain of the G protein, thereby blocking the virus from attaching to the CX3CR1 receptor on host cells (12). This *in vitro* antiviral effect of maoto was further confirmed in a murine model of RSV infection (12). These findings revealed that EH and CC exert unique antiviral activities against at least two distinct RNA viruses, employing different mechanisms of action.

In the case of influenza virus infection, it can be hypothesized that maoto exerts its antiviral effect through mechanisms other than the previously reported inhibition of the uncoating process. Here, we present evidence that EH and CC interact with hemagglutinin, the spike protein on the viral envelope, to block viral attachment to the cell membrane, similar to their antiviral action in RSV infection. Additionally, we found that the polymerase acidic (PA) protein within viral ribonucleoprotein (RNP) interacts with EH and CC, inhibiting the endonuclease activity of PA and subsequently reducing viral genome transcription. These findings show how maoto employs multiple antiviral mechanisms against influenza infection.

## RESULT

### Anti-infective activity of maoto in cultured cells in the early stages of the life cycle against several influenza viruses

**Fig 1:**
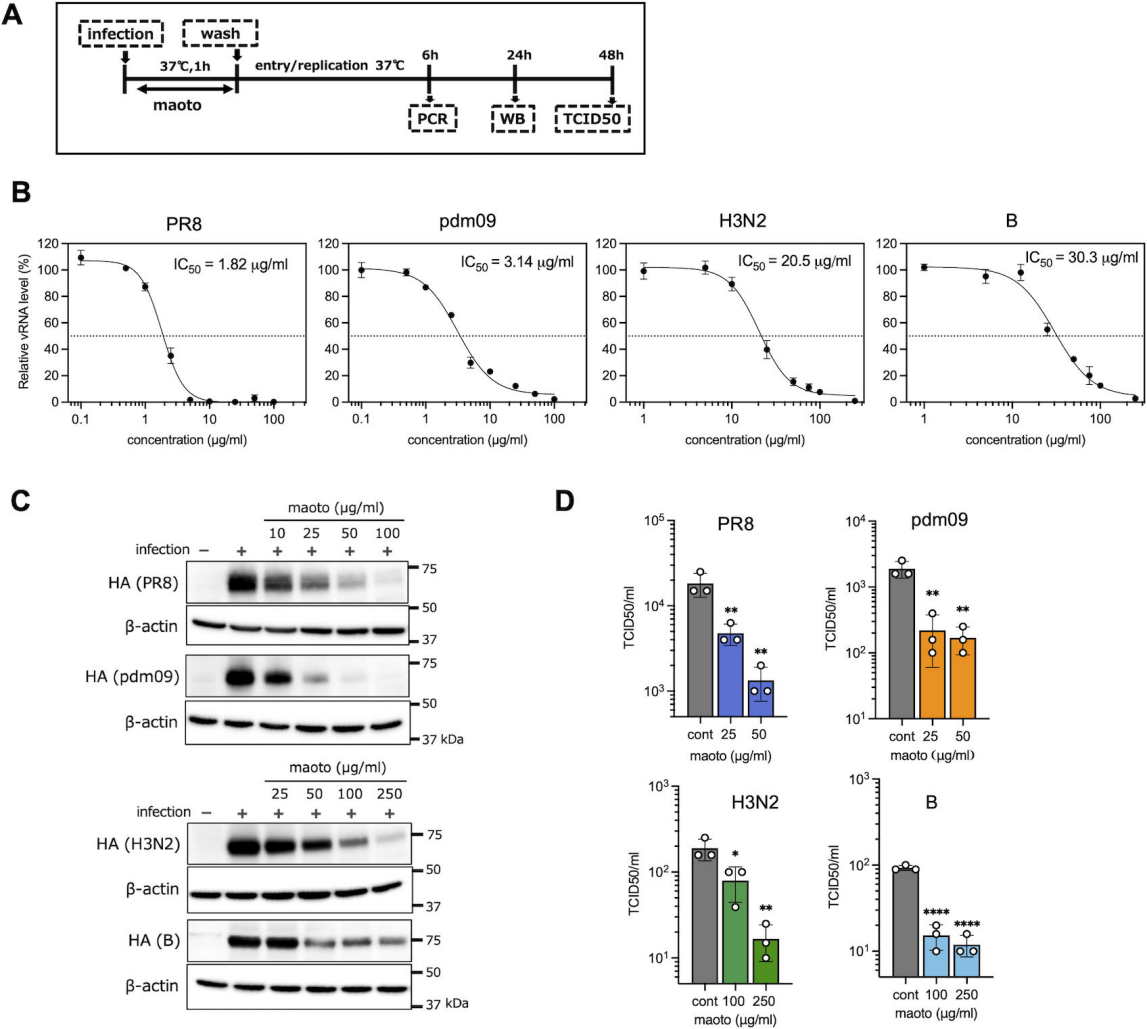
Anti-infective activity of maoto in cultured cells in the early stages of the life cycle of the tested influenza viruses. (**A**) Time schedule for sample collection for virus infection experiments. A549 cells were infected with the influenza virus (multiplicity of infection, MOI = 1) in the presence or absence of maoto at 37°C for 1 h, followed by incubation without virus and maoto for the indicated times. (**B**) Dose-dependent decrease of vRNA by maoto. The level of viral RNA in the cells after 6 h of incubation was quantified by RT-qPCR. IC_50_ was determined by logistic regression analysis. The graph shows mean ± error bars SD (*n* = 3). (**C**) Reduction of viral proteins by maoto. Whole-cell extracts were prepared from cells 24 h after infection of each of the viruses with various concentrations of maoto (10, 25, 50, 100 µg/mL for PR8 and pdm09, 25, 50, 100, 250 µg/mL for H3N2 and B), as described in A, and analyzed by western blotting using antibodies against the HA protein of each virus strain. (**D**) Virus titers after treatment with maoto. A549 cells were infected with the influenza viruses tested in the presence of maoto (25 and 50 µg/mL for PR8 and pdm09, 100 and 250 µg/mL for H3N2 and B), as described in A. The virus titer in supernatants 48 h after infection was determined by TCID50 assay. The graph shows individual values (circle) and mean (bar) ± error bars SD (*n* = 3). One-way analysis of variance (ANOVA) followed by the Dunnett post-test. *P < 0.05, **P < 0.01, and ****P < 0.0001.

The early stage of the influenza virus proliferation can be delineated into two distinct phases: the initial binding, where the virus attaches to the host cell, and subsequent steps involving invasion, transcription, and replication. To elucidate the molecular mechanism of anti-infective activity against the influenza virus, we investigated which phase of the infection process is disturbed by maoto. First, similar to our previous experiments with RS virus (12, 13), maoto was administered during the binding phase, and its antiviral efficacy was assessed based on the viral RNA, protein, and titer levels (Fig. 1A). The quantity of viral RNA in host cells was quantified at 6 h post-viral infection using quantitative reverse transcription PCR (RT-qPCR) of the hemagglutinin (HA) gene across several influenza viruses (A/H1N1/Puerto Rico [PR8], A/H1N1/California [pdm09], A/H3N2/Victoria [H3N2], and B/Brisbane [B]). Maoto exhibited dose-dependent antiviral activity against all of the tested influenza virus strains (Fig. 1B). The 50% inhibitory concentration (IC_50_) values for PR8 and pdm09, both influenza A virus H1N1, were 1.82 µg/mL and 3.14 µg/mL, respectively, demonstrating effectiveness at concentrations approximately one-tenth that required for A/H3N2 (20.5 µg/mL) and B (30.3 µg/mL). Viral proteins in host cells were detected 24 h after infection through western blotting (Fig. 1C). Consistent with the findings for viral RNA, the viral protein level within host cells was significantly reduced by maoto in a dose-dependent manner. Notably, PR8 and pdm09 exhibited greater reductions in viral protein levels at lower concentrations of maoto compared to H3N2 and B. Viral titers of the culture medium incubated for 48 h post-infection in the presence or absence of maoto were subsequently quantified using Median Tissue Culture Infectious Dose (TCID50) (Fig. 1D). The virus titer reduced significantly in the presence of maoto, suggesting that the production of infective progeny viruses was markedly inhibited by the action of maoto. Our results showed that the viral RNA and protein levels and virus titer were significantly decreased when maoto was present during the viral binding phase, indicating an antiviral effect in the early phase of the life cycle of the virus. Maoto exhibited variable antiviral activity across the various viruses.

### Binding of influenza virus hemagglutinin protein to maoto

**Fig 2:**
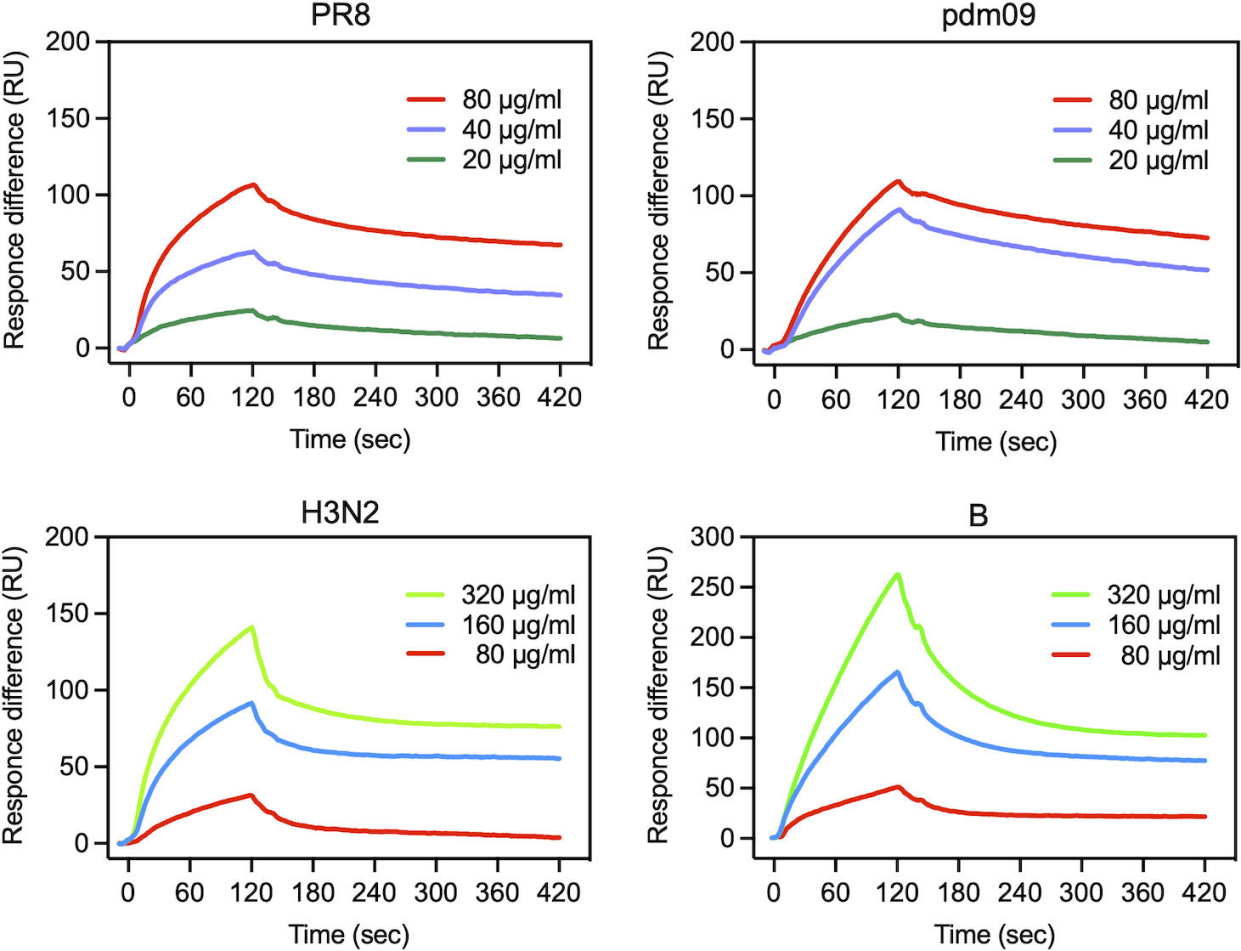
Binding of influenza virus hemagglutinin protein to maoto. Binding of maoto to influenza HA protein immobilized on a sensor chip by SPR. Three different concentrations of maoto (5, 10, 20 µg/mL for PR8 and pdm09, 80, 160, 320 µg/mL for H3N2 and B) were injected for 120 s.

We hypothesized that, similar to the mechanism observed with the RS virus, specific components of maoto would interact with glycoprotein (hemagglutinin) on the viral envelope, thereby inhibiting its binding to host cell receptors and effectively blocking the entry of the virus. To test this hypothesis, surface plasmon resonance (SPR) was employed to investigate the interaction between influenza virus hemagglutinin and maoto (Fig. 2). Hemagglutinin of the influenza virus was immobilized on a sensor chip, and maoto was added as the analyte. Although the binding concentration varied by virus type, we found a dose-dependent increase in resonance units with maoto, indicating the binding of maoto to hemagglutinin. Furthermore, it was confirmed that maoto did not easily dissociate from hemagglutinin, suggesting a strong and stable binding interaction. From the shape of the sensorgrams, it can be inferred that these viruses have different binding modes. The necessity of a higher concentration of maoto to achieve an equivalent response that was observed with H3N2 and B can be explained by differences in the amino acid sequence of hemagglutinin (Table S1).

### Inhibitory effect of maoto during the influenza virus entry and replication phases

**Fig 3:**
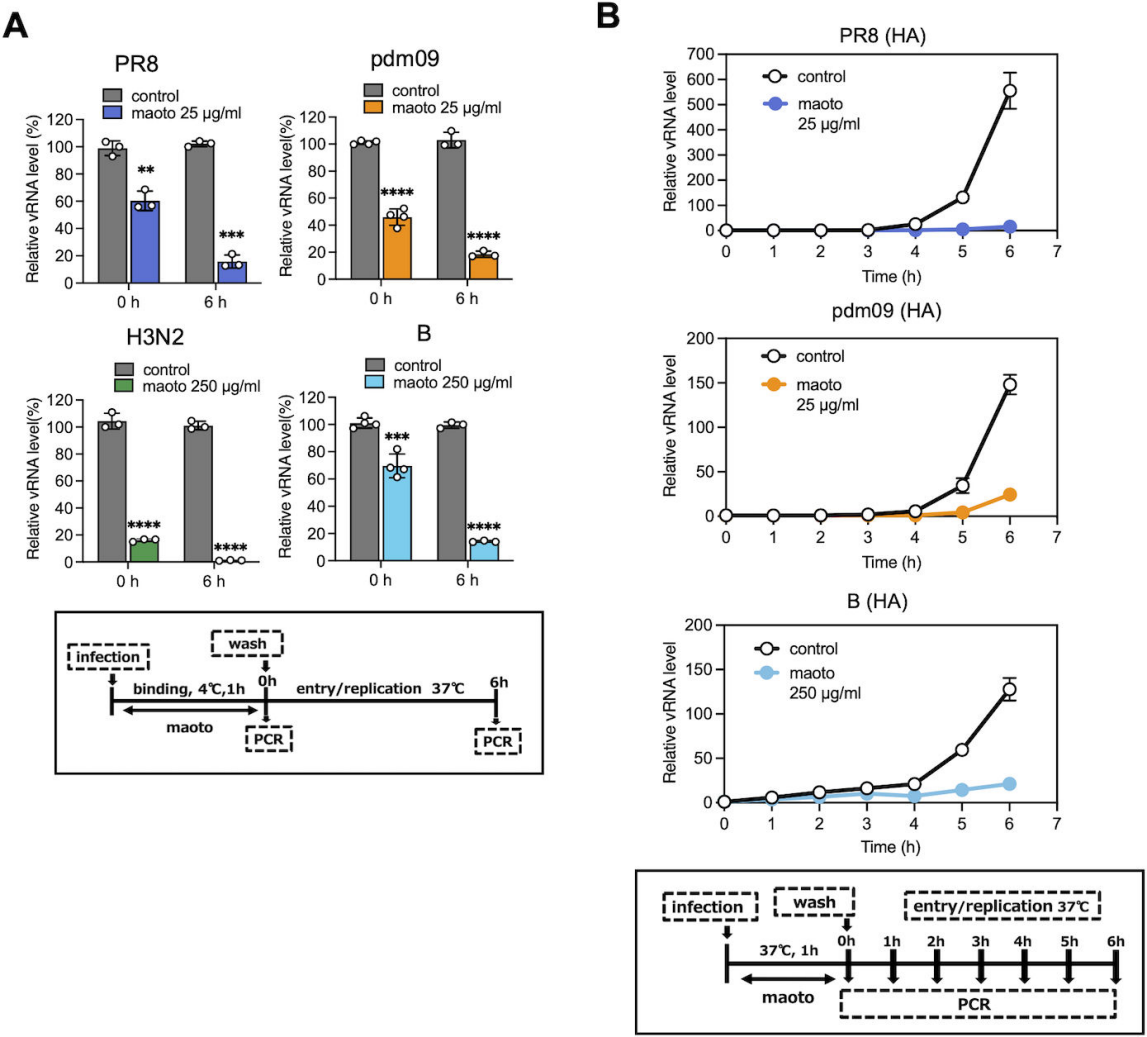
Inhibitory effect of maoto during the influenza virus entry and replication phases. (**A**) Comparison of quantities of viral RNA at 0 and 6 h after infection. A549 cells were infected with the influenza virus (MOI = 1) in the presence or absence of maoto (25 µg/mL for PR8 and pdm09, 250 µg/mL for H3N2 and B) at 4°C for 1 h, then total RNA was prepared immediately after infection and after 6 h incubation. Viral RNA levels were measured by RT-qPCR. The graph shows individual values (circle) and mean (bar) ± error bars SD (*n* = 3). Statistical significance was determined using an unpaired Student’s *t*-test. **P < 0.01, ***P < 0.001, and ****P < 0.0001. (**B**) Viral replication with or without maoto treatment (25 µg/mL for PR8 and pdm09, 250 µg/mL for B). Virus-infected cells were harvested hourly, as shown in the graphs, and vRNA was quantified with qPCR using HA gene primers. The data from qPCR are shown as a line graph with time 0 h as 1. The graph shows mean ± error bars SD (*n* = 3).

To confirm that maoto inhibits viral binding to host cells, we used RT-qPCR to quantify the amount of virus on the host cell surface. Since cellular activities are reduced at low temperatures, viruses remain bound to cell receptors without entering the cell (16). Utilizing this characteristic, we infected cells with the virus at 4°C, followed by quantification of viral RNA on the cell surface (Fig. 3A). Based on the previously determined efficacy of maoto shown in Fig. 1B different concentrations of maoto were administered depending on the virus strain: 25 µg/mL for PR8 and pdm09 and 250 µg/mL for H3N2 and B. The viral RNA level was significantly reduced in H3N2. Unexpectedly, the reduction in the viral RNA levels of PR8, pdm09, and B was less than 50% at 0 h compared to untreated controls. When the viral RNA level was quantified after 1 h of infection at 4°C, followed by 6 h incubation at 37°C, the amount of viral RNA in all strains was reduced to less than 20% that of untreated controls. These results suggest that maoto not only inhibits viral binding to hemagglutinin and its entry into the host cell but also exerts an effect on virus post-entry, as evidenced by the reduction of the virus during the 6 h intracellular period. To explore the post-entry effect in detail, intracellular viral RNA levels were monitored every hour post-infection by RT-qPCR (Fig. 3B). In the absence of maoto, rapid viral multiplication was observed starting 4 h after infection, but this was not observed in the presence of maoto. Comparable results were obtained using a different primer set for RT-qPCR (Fig. S1). The addition of maoto at 3 h post-infection, after viral entry into host cells, did not significantly alter viral RNA levels (Fig. S2). Additionally, treatment with maoto after the virus had invaded the cells did not exhibit strong antiviral activity at pre-infection concentrations (Fig. S3). These results indicate that maoto enters the cytosol concurrently with viral entry and that it suppresses intracellular viral replication.

### Anti-infective activity of maoto components in cultured cells during the early stages of the influenza virus life cycle and binding of the components to influenza virus HA protein

**Fig 4:**
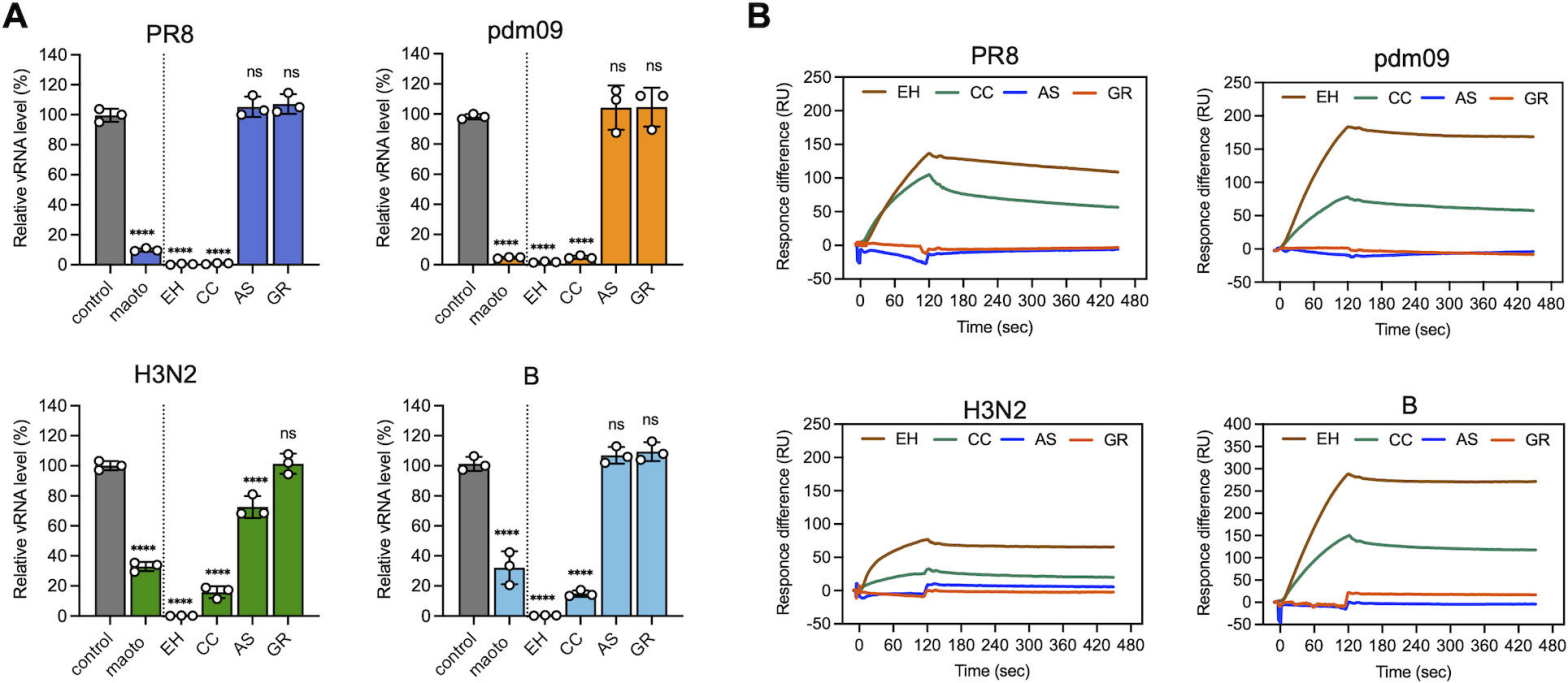
Anti-infective activity of maoto components in cultured cells during the early stages of the influenza virus life cycle and binding of the components to influenza virus HA protein. (**A**) A549 cells were infected with the influenza virus (MOI = 1) at 37°C for 1 h in the presence or absence of 50 µg/mL of maoto and of 10 µg/mL of the maoto components EH, CC, AS, and GR. The viral RNA level of cells prepared after 6 h incubation was quantified by RT-qPCR using HA gene primers. The graph shows relative viral RNA amounts compared to the control with individual values (circle) and mean (bar) ± error bars SD (*n* = 3). Statistical significance was determined using one-way ANOVA followed by Dunnett’s post-test. ****P < 0.0001; ns, not significant. (**B**) The binding of maoto components (10 µg/mL for EH, CC, AS, and GR) to influenza HA protein immobilized on a sensor chip was measured by SPR. Maoto components were injected for 120 s.

The antiviral activity of the four herbal crude drugs (EH, CC, AS, and GR) that are components of maoto was evaluated. They were added simultaneously with infection, and the intracellular viral RNA level was quantified by RT-qPCR 6 h after infection (Fig. 4A). EH and CC exhibited a potent antiviral effect, whereas AS and GR showed negligible activity. Next, we determined the IC_50_ values for the antiviral effect of EH and CC (Table 1; Fig. S4). Both EH and CC demonstrated greater activity against PR8 and pdm09 than against H3N2 and B. Furthermore, EH was more effective than CC against all four influenza viruses. The CC₅₀ values of EH and CC were significantly higher than their IC₅₀ values, suggesting that their antiviral activity is not attributable to cytotoxic effects on host cells (Table 1). The interaction between hemagglutinin and the crude drugs was subsequently validated using SPR (Fig. 4B). Similar to the findings depicted in Fig. 4A, EH and CC exhibited a robust interaction, whereas AS and GR showed no discernible interaction, which indicates that EH and CC bind to hemagglutinin, thereby inhibiting cellular entry.

**TABLE 1:** IC_50_ values for influenza viruses, the CC_50_ values for A549 cells, and SI values^*a*^

Influenza virus	Strain	IC_50_ (µg/mL)		CC_50_ (µg/mL)		SI	
		EH	CC	EH	CC	EH	CC
A(H1N1) PR8	A/Puerto Rico/8/34	0.63	0.88	406.8	557.4	645.7	633.4
A(H1N1) pdm09	A/California/7/2009	0.76	1.33			535.2	419.1
A(H3N2)	A/Victoria/210/2009	1.71	2.75			237.8	202.6
Influenza B	B/Brisbane/60/2008	2.13	3.90			190.9	142.9

^*a*^ IC50 for influenza viruses: a dose-dependent reduction in vRNA was observed with the EH and CC treatments. The vRNA level of cells 6 h post-infection was quantified using RT-PCR. CC50 for A549 cells: A549 cells were seeded in 96-well plates and treated with EH (100–1,000 µg/mL) or CC (100–1,000 µg/mL). Cell viability was measured 72 h post-treatment with the MTS assay. The IC50 values for the tested influenza viruses and the CC50 values for A549 cells were determined through logistic regression analysis. The selectivity index (SI) was calculated by dividing CC50 by IC50. The data were obtained from at least three experiments.

### Inhibitory effect of EH and CC during the influenza virus PR8 entry and replication phases

**Fig 5:**
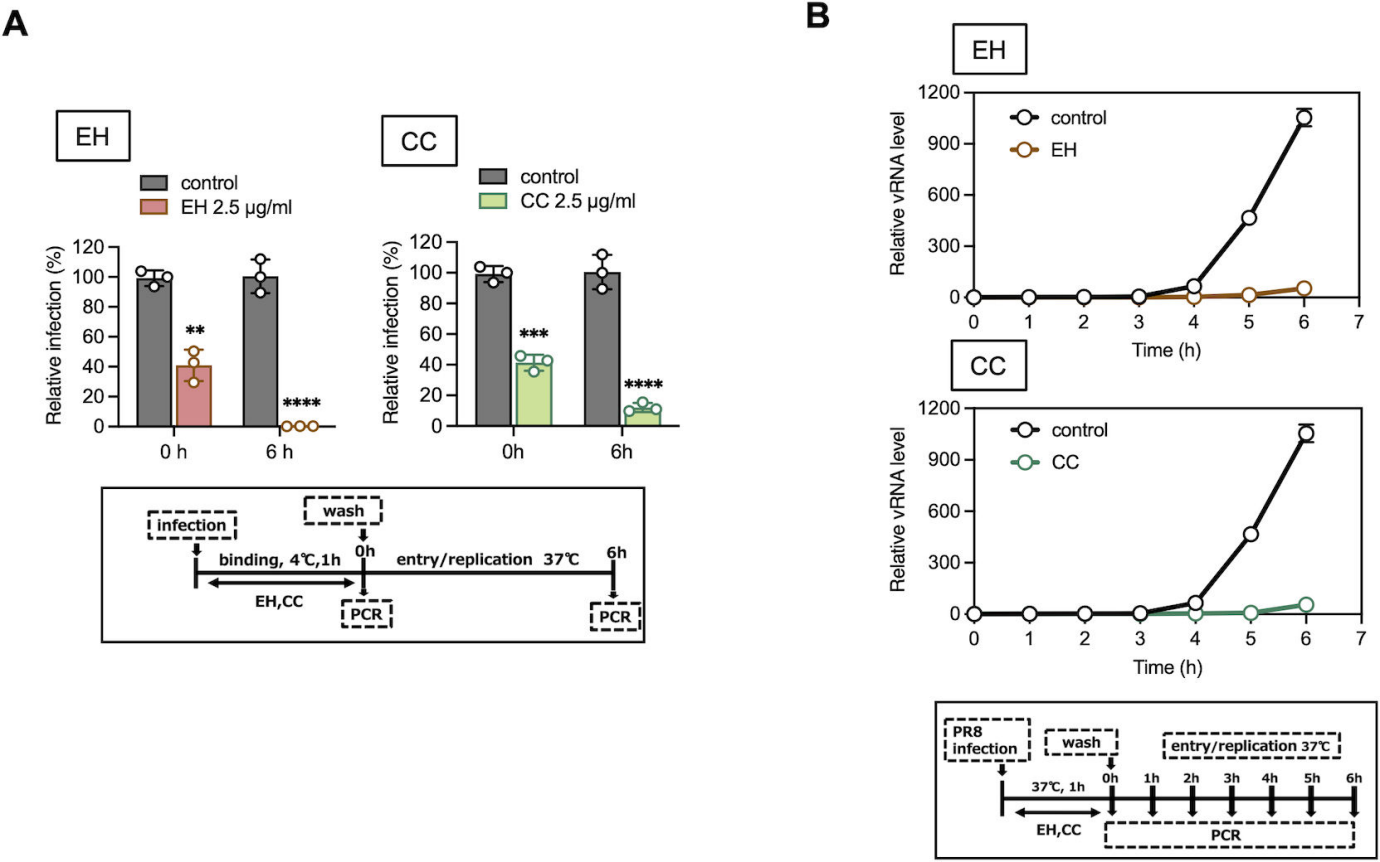
Inhibitory effect of EH and CC during the influenza virus PR8 entry and replication phases. (**A**) Comparison of the quantity of viral RNA at 0 and 6 h after infection. A549 cells were infected with the influenza virus H1N1 PR8 (MOI = 1) in the presence or absence of EH (2.5 µg /mL) or CC (2.5 µg/mL) at 4°C for 1 h, then total RNA was prepared immediately after infection and after 6 h incubation, as shown in the graphs. The viral RNA level was measured by RT-qPCR using HA gene primers. The graph shows individual values (circle) and mean (bar) ± error bars SD (*n* = 3). Statistical significance was determined using unpaired Student’s *t*-test. **P < 0.01, ***P < 0.001, and ****P < 0.0001. (**B**) Viral replication with or without EH or CC treatment. Virus-infected cells were harvested hourly, as shown in the graphs, and vRNA was quantified with qPCR using HA gene primers. The data from qPCR is shown as a line graph with the 0 h time point as 1. The graph shows mean ± error bars SD (*n* = 3).

Virus on the surface of host cells infected with PR8 for 1 h at 4°C in the presence of EH and CC was quantified for viral RNA using RT-qPCR. Intracellular viruses were also quantified and compared after 6 h of incubation (Fig. 5A). The concentrations of EH and CC utilized in this study were adjusted according to the data presented in Fig. S4. The results indicate that, similar to the case of maoto, the number of viruses on the cellular surface at the end of infection (0 h) was reduced by EH and CC treatment; however, the decrease was not as effective as that observed in Fig. 4A. In contrast, intracellular viruses at 6 h post-infection were significantly diminished (Fig. 5A). This aligns with the findings observed with maoto (Fig. 3A). The amount of viral RNA after PR8 infection at 37°C for 1 h increased from 4 to 6 h post-infection, whereas production of viral RNA was totally suppressed in the presence of EH or CC (Fig. 5B). This suggests that constituents of maoto, such as EH and CC, not only inhibit viral binding to cells, but the constituents incorporated into the cells exert a suppressive effect on intracellular viruses.

### Inhibition of influenza A virus PA endonuclease activity by maoto and maoto components

**Fig 6:**
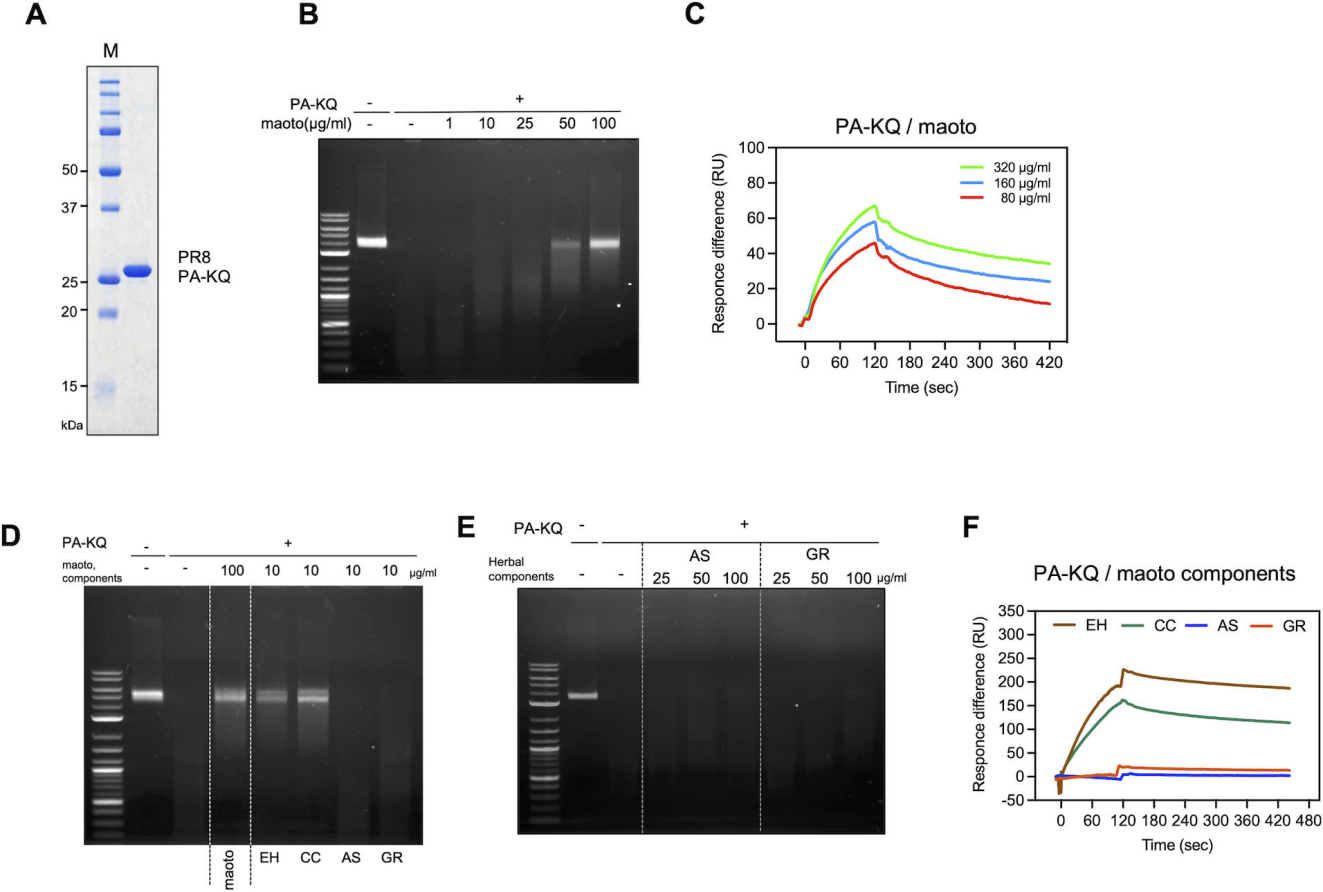
Inhibition of influenza A virus PA endonuclease activity by maoto and maoto components. (**A**) Preparation of acetylation mimic mutant PA protein (PA-KQ). PA-KQ protein was produced in *Escherichia coli* and purified homogeneously. The purified protein was subjected to SDS-PAGE and visualized by Coomassie Brilliant Blue staining. (**B**) PA-KQ endonuclease activity assay. The single-stranded M13mp18 DNA was incubated with or without PA-KQ in the presence of various concentrations of maoto (1, 10, 25, 50, and 100 µg/mL) and analyzed by agarose gel electrophoresis, followed by EtBr staining. (**C**) The binding of maoto to PA-KQ protein immobilized on a sensor chip was assessed by SPR. Maoto (80, 160, and 320 µg/mL) was injected for 120 s. (**D**) Inhibition of PA-KQ endonuclease activity assay. Single-stranded M13mp18 DNA was incubated with or without PA-KQ in the presence of the maoto components (10 µg/mL). The samples were analyzed by 0.8% agarose gel electrophoresis, followed by EtBr staining. (**E**) No inhibitory activity of AS and GR. The PA-KQ activity in the presence of AS and GR (25, 50,100 µg/mL) was analyzed, as described in (**B**). (**F**) The binding of maoto components (EC, CC, AS, and GR) to PA-KQ protein immobilized on a sensor chip was assessed by SPR. Maoto components (20 µg/mL) were injected for 120 s.

Our results demonstrated that maoto binds to influenza hemagglutinin and inhibits virus entry into host cells (Fig. 3A). Additionally, the significant suppression of virus increases from 4 to 6 h post-infection suggests that maoto may inhibit viral transcription and replication, which are catalyzed by influenza virus heterotrimeric RNA-dependent RNA polymerase (RdRp) consisting of PA, PB1, and PB2, which are responsible for endonuclease, RNA polymerase, and 5′ cap binding, respectively. The PA contains an endonuclease domain in its N-terminus that is critical for cleaving mRNA caps to initiate viral mRNA transcription (17–19). Therefore, we investigated whether maoto can inhibit the enzymatic activity of the endonuclease domain. We prepared recombinant PR8 PA endonuclease domain (1–200 amino acids) with an acetylation-mimic K19Q mutation (PA-KQ) that has enhanced endonuclease activity (Fig. 6A) (20). The recombinant PA-KQ degraded the substrate M13mp18 ssDNA completely in the absence of maoto (21–24); however, degradation was inhibited in a dose-dependent manner by maoto (Fig. 6B). Inhibition was evident at concentrations of 50–100 µg/mL, which is similar to the concentration range for interaction with the PA-KQ protein as analyzed by SPR (Fig. 6C). Next, we investigated which maoto components were responsible for the inhibitory activity. The substrates remained intact in the presence of 5–10 µg/mL of EH and CC (Fig. 6D; Fig. S5C and D), whereas AS and GR had little inhibitory activity, even at higher concentrations (Fig. 6E). SPR analysis showed that EH and CC, but not AS and GR, interacted with PA-KQ protein, which explains their nuclease inhibitory activity (Fig. 6F). At the tested concentrations, maoto itself and its individual components had no nuclease activity (Fig. S5A and B). These results suggest that EH and CC in maoto are responsible for the inhibitory effect on nuclease activity.

## DISCUSSION

Maoto has been used clinically for the treatment of respiratory infectious diseases since ancient times. In this study, we found that it exerts two distinct antiviral mechanisms against the influenza virus. First, it binds to the HA glycoprotein, thereby inhibiting viral entry into host cells. Influenza virus infection is initiated by the binding of HA to sialic acid receptors on host cells, positioning HA as a promising target for drug development (25–28). However, its structural complexity has impeded detailed characterization, preventing the development of HA-targeted drugs (29). To date, no effective anti-influenza drugs specifically targeting HA have been developed. Maoto, with its diverse components, presents a potential solution to overcoming these challenges in developing HA-targeted therapeutic drugs. The arrays of compounds in maoto suggest the presence of compounds with specificity for HA’s complex structure, enabling high-affinity binding (30). The observed variations in maoto’s effective concentrations and the distinct sensorgram patterns detected in SPR among different HA subtypes may be attributed to the specificity of maoto components, likely modulated by the HA amino acid sequence (Fig. 2). This implies that maoto has the potential to adapt flexibly to viral mutations.

Second, maoto interacts with the PA subunit of heterotrimeric RdRp, composed of the PA, PB1, and PB2 subunits of the influenza virus, and suppresses CAP-dependent endonuclease activity (17–19). The endonuclease activity of PA is a promising target for novel therapeutics, as it plays a pivotal role in viral replication (31, 32). Currently, RNA polymerase inhibitors such as baloxavir marboxil (33) and favipiravir (34, 35) target the cap-dependent nuclease activity in transcription and the RNA-dependent RNA polymerase activity in transcription/replication, respectively, to suppress the amplification of vRNP. Baloxavir marboxil is available as an RNA polymerase inhibitor, but its use in children is discouraged due to a higher viral mutation rate in children (36–40). Moreover, while favipiravir poses a lower risk of inducing drug resistance, it has demonstrated teratogenic effects in animal studies (41, 42), making it unsuitable for the treatment of seasonal influenza virus infections (43). The observation that maoto inhibited endonuclease activity in this study is a promising outcome, suggesting its potential as a novel polymerase inhibitor. Notably, the addition of Maoto to cells already infected with the virus did not significantly alter the intracellular virus levels. (Fig. S2 and S3), the simultaneous presence of maoto during infection effectively inhibited proliferation (Fig. 3A). This implies that maoto may be internalized by cells alongside the virus. Because the influenza virus enters cells via endocytosis, it is plausible that maoto bound to the virus co-enters into endosomes. Our previous investigation into the antiviral activity of maoto against the RS virus demonstrated a limited effect on intracellular virus populations (12), suggesting that its antiviral activity operates through distinct mechanisms depending on the virus. This makes maoto particularly intriguing, as it demonstrates multiple modes of action against influenza viruses. Treatment of severe influenza patients with maoto and other anti-influenza drugs may be more effective than treatment with conventional drugs alone.

**Fig 7:**
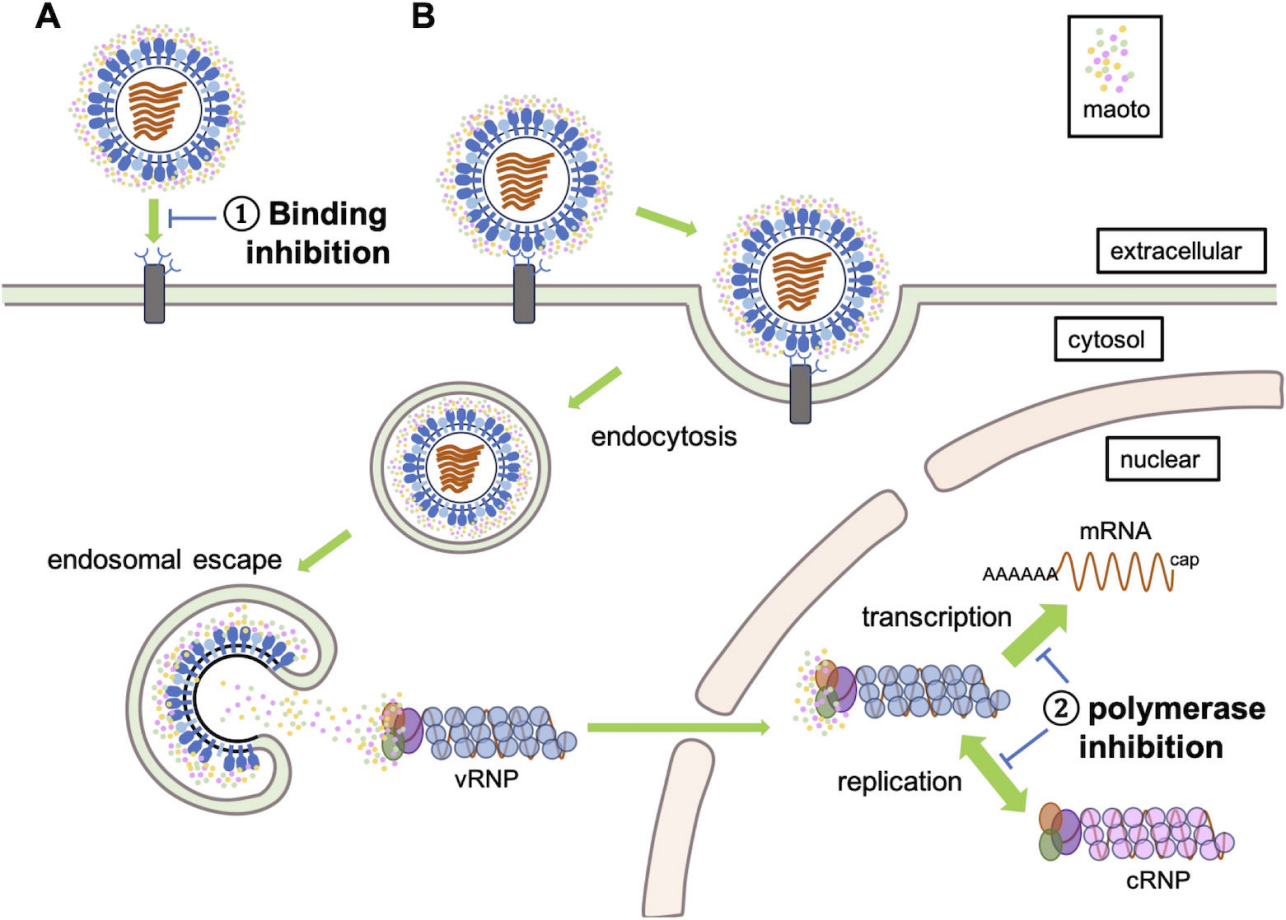
Model for the mode of the actions of maoto against influenza virus infection. (**A**) Mechanism of inhibition of viral binding to host cells. Maoto binds to viral hemagglutinin, blocking its interaction with the host cell receptor sialic acid, thereby preventing infection. (**B**) Mechanism of action after viral entry. When the virus binds to host cells, maoto is internalized with the virus into endosomes through endocytosis. During uncoating, maoto binds to the polymerase of vRNPs, which subsequently translocates into the nucleus. This interaction inhibits viral transcription and replication within the nucleus.

As shown above, maoto is co-internalized into the endosome through endocytosis along with the influenza virus. Maoto may bind to the vRNPs PA, PB1, or PB2 during the virus uncoating process. These maoto-bound vRNPs are subsequently transported to the nucleus, where, due to the inhibition of the endonuclease activity of PA by maoto, mRNA synthesis is halted, disrupting transcription. Moreover, the interaction of maoto with PB1 and PB2 possibly impairs their polymerase activity, thereby inhibiting cRNA synthesis and subsequently suppressing virus genome replication. We propose this as a potential mechanism of the action for maoto (Fig. 7). The precise timing of maoto interaction with vRNPs and its binding to PB1 and PB2 remains unclear, which will necessitate further experimental validation.

Maoto exhibits unique antiviral properties by targeting multiple mechanisms of action, indicating its broad-spectrum efficacy against various viral mutations. These insights will contribute to developing novel antiviral drugs and enhancing therapeutic strategies. Among the crude drugs maoto, both EH and CC demonstrated antiviral activity. The primary compounds in EH are ephedrine alkaloids, which stimulate the nervous system and are associated with side effects such as palpitations, hypertension, insomnia, and dysuria (44, 45). However, the EH component responsible for anti-influenza virus activity is distinct from ephedrine alkaloids (46). The same applies to its activity against the respiratory syncytial (RS) virus (13). We have identified several non-ephedrine alkaloid components in EH that exhibit potent anti-RS virus activity. These components also demonstrate strong antiviral activity against the influenza virus, and we plan to analyze their interactions with hemagglutinin and PA in the future. Additionally, we are working to identify the active components of CC and anticipate discovering compounds distinct from those in EH.

## MATERIALS AND METHODS

### Cell lines

Human lung carcinoma (A549) cells and Madin-Darby canine kidney (MDCK) cells were purchased from the American Type Culture Collection (ATCC, Manassas, VA) and maintained in Dulbecco’s modified Eagle medium (DMEM, Fujifilm, Tokyo, Japan) supplemented with 10% fetal bovine serum (FBS, CORNING, US) and 1% penicillin-streptomycin (Fujifilm, Tokyo, Japan). The cultures were maintained at 37°C in a 5% CO_2_ atmosphere.

### Viruses

Influenza A/PuertoRico/8/34 (H1N1, PR8) was obtained from the Chemo-Sero-Therapeutic Research Institute (Kumamoto, Japan). Influenza A/California/07/2009 (H1N1, pdm09), A/Victoria/210/2009 (H3N2), and influenza B/Brisbane/60/2008 (B) were obtained from the National Institute of Infectious Diseases (Tokyo, Japan). Viruses were initially propagated in MDCK cells for 48 h at 37°C in serum-free D-MEM containing trypsin (2 µg/mL). Virus-containing culture supernatants were stored at −80°C until use. Virus titers were determined by the 50% Tissue Culture Infectious Dose assay (TCID50) using MDCK cells.

### Antiviral reagents

Maoto, supplied by TSUMURA & CO. (Tokyo, Japan), is an extract derived from four plants: Ephedrae Herba (ephedra; EH), Cinnamomi Cortex (cinnamon; CC), Armeniacae Semen (apricot; AS), and Glycyrrhizae Radix (licorice; GR). The components were extracted in water by boiling at a ratio of 10 (EH):10 (CC):8 (AS):3 (GR). Maoto powder was prepared by concentration and spray-drying of the decoction. Prior to experimental use, the Maoto was dissolved in warm PBS and incubated for 1 h. After sedimentation at 3,000 rpm, the supernatant was collected and filtered using a 0.45 µm membrane filter. The prepared Maoto solution was stored at −80°C until use.

### Real-time PCR analysis

A549 cells (1 × 10⁵) were seeded into 24-well plates and infected with influenza virus (multiplicity of infection, MOI = 1) supplemented with Maoto (0–250 µg/mL) at 4°C (for detecting virus on cellular surface) or 37°C for 1 h. Following infection, the virus solution was removed, and the cells were washed with PBS before being incubated in fresh medium at 37°C. At the appropriate timing after post-incubation, the cells were washed again with PBS, and total RNA was extracted using ISOGEN II (Nippon Gene, Tokyo, Japan). cDNA was synthesized using the PrimeScript RT reagent kit with gDNA eraser (Takara Bio, Shiga, Japan), following the manufacturer’s protocol. Quantitative PCR was performed using the CFX Connect system (Bio-Rad) as described previously (12). Briefly, cDNA was amplified using THUNDERBIRD qPCR mix (Toyobo, Osaka, Japan) with primers for human β-actin 5′-TGGCACCCAGCACAATGA-3′ and 5′-CTAAGTCATAGTCCGCCTAGAAGCA-3′.

Influenza A/PuertoRico/8/34 HA region 5′-CCTGCTCGAAGACAGCCACAACG-3′ and 5′-TTCCCAAGATCCATCCGGCAA-3′. PA region 5′-GCTTCTTATCGTTCAGGCTCTTAGG-3′ and 5′-CCGAGAAGCATTAAGCAAAACCCAG-3′. Influenza A/California/07/2009 HA region 5′-GGAGCAAAAAGCTTCTACAA-3′ and 5′-ACTTTGTTGGTCAGCACTA-3′. NA region 5′-GACAGGCCTCATACAAGATCTTC-3′ and 5′-TGCCAGTTATCCCTGCACACACA-3’. Influenza A/Victoria/210/2009 HA region 5′-CTATTGGACAATAGTAAAACCGGGRGA-3′ and 5′-GTCATTGGGRATGCTTCCATTTGG-3′. Influenza B/Brisbane/60/2008 HA region 5′-GGGGGGAGCAGAAGCAGAGC-3′ and 5′-CCGGGTTATTAGTAGTAACAAGAGC-3′. NS region 5′-GGAGCAACCAATGCCAC-3′ and 5′-GTKTAGGCGGTCTTGACCAG-3′.

The reaction consisted of 40 cycles of denaturation at 95°C for 15 s and annealing/extension at 60°C for 1 min. Data were analyzed using CFX3.1 software (Bio-Rad). Viral inhibition was assessed by setting the viral activity without the drug as the negative control well to 100% and calculating the percentage reduction for each Maoto concentration. The 50% effective concentration (IC50) was determined using a log versus response logistic nonlinear regression equation in GraphPad Prism 10 software (GraphPad Software, San Diego, CA, USA).

### Antibodies

Anti-influenza A/PuertoRico/8/34 HA (11684-T62) antibody was purchased from Sino Biological (Beijing, China). Anti-influenza A/California/07/2009 HA (GTX127357), anti-influenza A/Victoria/210/2009 (H3N2) HA (GTX127363), and anti-influenza B/Brisbane/60/2008 HA (GTX128542) antibodies were purchased from Gene Tex (Irvine, CA). Anti-β-actin (010-27841) antibody was obtained from Fujifilm (Tokyo, Japan).

### Western blotting analysis

A549 cells (1 × 10^5^) were seeded in 24-well plates and subsequently infected with influenza virus (MOI = 1) supplemented with maoto (10, 25, 50, 100 µg/mL for PR8 and pdm09 strains, and 25, 50, 100, 250 µg/mL for H3N2 and B strains) at 37°C for 1 h, followed by removing the viral solution with aspiration. After washing with PBS, the cells were incubated at 37°C in fresh medium for 24 h and harvested in a lysis buffer (100 mM Tris-HCl [pH 6.8], 2% SDS, 20% glycerol, 2% β-mercaptoethanol, and 0.4 mg/mL bromophenol blue), and boiled at 100°C for 20 min. Ten micrograms of soluble proteins were separated by SDS-PAGE and transferred to a PVDF membrane (Bio-Rad) by Trans-Blot Turbo (Bio-Rad). The membrane was blocked with Can Get Signal PVDF Blocking Reagent (Toyobo) for 1 h, then incubated overnight with the following primary antibodies: anti-influenza A/PuertoRico/8/34 HA antibody (1:1,000, 11684-T62; Sino Biological), anti-influenza A/California/07/2009 HA antibody (1:1,000, GTX127357; Gene Tex), anti-influenza A/Victoria/210/2009 (H3N2) HA antibody (1:1,000, GTX127363; Gene Tex), anti-influenza B/Brisbane/60/2008 HA antibody (1:1,000, GTX128542; Gene Tex), and anti-β-actin (1:5,000, 010-27841; Fujifilm) antibody. After washing with PBS containing 0.1% Tween 20, the membrane was incubated with HRP-conjugated secondary antibody (1:5,000, NA934V, NA931V; Cytiva, US) followed by detection with SuperSignal West Pico PLUS Chemiluminescent Substrate (Thermo Fisher, MA, US) by LAS-3000 (GE Healthcare).

### Virus yield reduction assay (TCID50)

A549 cells (1 × 10^5^) were plated in 24-well plates and infected with influenza virus (MOI = 1) mixed with maoto (25 and 50 µg/mL for PR8 and pdm09 strains, 100 and 250 µg/mL for H3N2 and B strains) at 37°C for 1 h. The virus solution was then removed, the cells were washed with PBS, and then incubated at 37°C in fresh medium for 48 h. The supernatants were collected, and the virus titer was determined by the 50% Tissue Culture Infectious Dose (TCID50) assay on MDCK cells.

### Surface plasmon resonance

Recombinant C-terminal 6x His-tagged influenza H1N1 PR8 HA protein (A42599) and influenza H1N1 pdm09 HA protein (PR-88193) were purchased from Thermo Fisher. Recombinant C-terminal 6x His-tagged influenza H3N2 HA protein (HA2-V52H3) was purchased from Acro Biosystems. Recombinant C-terminal 6x His-tagged influenza B HA protein (40016-V08H) was purchased from Sino Biological. C-terminal 6x His-tagged PR8 PA-KQ protein was produced in *Escherichia coli* and purified as described below.

The ligands, diluted in PBS, were immobilized on a Sensor Chip NTA (Cytiva). Subsequent to PBS washing, maoto and its components, also diluted in PBS, were injected at a flow rate of 30 µL/min for 120 s to allow for association. The data were acquired using the Biocore J system (Cytiva) and analyzed with Biaevaluation software (Cytiva). All experimental procedures were conducted at a temperature of 25°C.

### Cytotoxicity and cell viability assay (CC_50_)

A549 cells (2.5 × 10^3^) were plated in 96-well plates and treated with EH (100–1,000 µg/mL) and CC (100–1,000 µg/mL) in 10% FBS-containing D-MEM. Cell viability was measured with the MTS assay at 72 h post-treatment, following the manufacturer’s instructions (Promega, Madison, Wisconsin, USA). The values of viability were determined as follows: the metabolic activity of the negative-control wells (with no drug) was set at 100%, and the percentage of reduction was calculated for each EH and CC concentration. The 50% cytotoxic concentration (CC₅₀) was calculated using the log versus response logistic nonlinear regression equation in GraphPad Prism 10 software (GraphPad Software, San Diego, CA, USA).

### Cloning of the PA endonuclease

DNA fragment encoding cap-dependent endonuclease domain (1–200 aa) of PA protein from the influenza H1N1 PR8 strain was prepared by PCR using oligonucleotides, 5′-AAAGCTAGCATGGAAGATTTTGTGCGACAATGCTTC-3′ and 5′-TTTTCTCGAGTTATTGGTCGGCAAGCTTGCGCATTG-3′ as primers, with cDNA derived from reverse transcription of the total RNA of cultured cells infected with H1N1 PR8 as a template. PCR product was cloned into pET28a (Novagen) using the NheI and XhoI sites, resulting in a pPA-C-His vector. To construct the acetylation mimic mutant PA nuclease (PA-KQ), we substituted the lysine residue at position 19 with glutamine residue using site-directed mutagenesis with primers 5′-GCTTGCGGAACAAACAATGAAAGAGTATG-3′ and 5′-TCGACAATCATCGGATTGAAGCATTGTCG-3′ with pPA-C-His vector as a template, resulting in a pPA-KQ-C-His vector.

### Production of recombinant protein

The pPA-KQ-C-His vector was transformed into BL21-CodonPlus (DE3)-RIPL (Agilent Technologies) cells. The cells were cultured in LB medium at 37°C until OD600 reached 0.4, at which point 0.5 mM IPTG was added to induce protein expression. The cells were further cultured at 18°C for 20 h, then collected by centrifugation at 4,000 × *g* for 10 min. The cell pellet was suspended in Buffer A (20 mM Tris-HCl, pH 8.0) and treated with 20 mg/mL lysozyme (Sigma-Aldrich) on ice for 10 min, followed by adding 0.5% Triton X-100 and sonication. After centrifugation, the soluble proteins were diluted in Buffer B (20 mM Tris-HCl, pH 8.0, 500 mM NaCl, and 20 mM imidazole) and subjected to a His Trap HP column (Cytiva) on AKTA explorer 10S (Cytiva) with 0–500 mM imidazole gradient over 10 column volumes. Proteins eluting at 130 mM imidazole were then diluted fivefold in Buffer C (50 mM Tris-HCl, pH 8.0, 10% glycerol) and further purified using a MonoQ 5/5 column (Cytiva) with 0–1 M NaCl gradient over 15 column volumes. The PA-KQ protein, eluted at 250 mM NaCl, was analyzed with SDS-PAGE, visualized by Coomassie Brilliant Blue staining, confirming homogeneity of 95%.

### Endonuclease inhibition analysis

The recombinant protein PA-KQ endonuclease assay was performed in 25 mM Tris-HCl (pH 8.0), 2.5 mM MnCl_2_, 1 mM DTT, 10% glycerol, 300 ng circular single-stranded DNA (M13mp18, Takara) as a substrate, and 250 ng recombinant PA endonuclease protein. After 1 h of digestion at 37°C, the samples were analyzed by 0.8% agarose gel electrophoresis and visualized by ethidium bromide staining to investigate the stability of ssDNA. Maoto and its components (EH, CC, AS, and GR) at various concentrations were preincubated with the PA-KQ endonuclease in the reaction mixture at room temperature.

### Statistical analysis

All data are presented as the mean ± standard error from at least three independent experiments. Statistical significance for comparisons among three or more groups was determined using analysis of variance (ANOVA) with Dunnett’s post-test, while comparisons between two groups were assessed using a paired or unpaired Student’s *t*-test. If the data did not follow a normal distribution, the Mann-Whitney U test was used. The significance of IC₅₀ and CC₅₀ values was determined using logistic regression analysis. Statistical significance was defined as follows:

****P < 0.001, ***P < 0.001, **P < 0.01, and *P < 0.05 versus control. Graph generation was conducted using GraphPad Prism 10 (GraphPad Software, CA, USA).

## SUPPLEMENTAL MATERIAL

Supplemental material spectrum.00371-25-s0001.pdf Table S1; Fig. S1 to S5.

ASM does not own the copyrights to Supplemental Material that may be linked to, or accessed through, an article. The authors have granted ASM a non-exclusive, world-wide license to publish the Supplemental Material files. Please contact the corresponding author directly for reuse.
