# Supplementary material for: Multiple antiviral mechanisms of Ephedrae Herba and Cinnamomi Cortex against influenza: inhibition of entry and replication
Source: Microbiol Spectr. 2025 Apr 30;13(6):e00371-25. doi: 10.1128/spectrum.00371-25 (PMC12131806; doi:10.1128/spectrum.00371-25)
Supplement: Supplemental material — Table S1; Fig. S1 to S5. [file spectrum.00371-25-s0001.pdf]

Supplementary Table 1. Amino acid homology between PR8 HA and HA of the different influenza virus subtypes.

Influenza virus subtype	Strain	Region	Amino acid identity to A(H1N1) Puerto Rico/8/34(PR8) HA (%)
A(H1N1)pdm09	A/California/7/2009	HA	81.2 %
A(H3N2)	A/Victoria/210/2009	HA	38.4 %
Influenza B	B/Brisbane/60/2008	HA	23.0 %

supplemental data fig.1

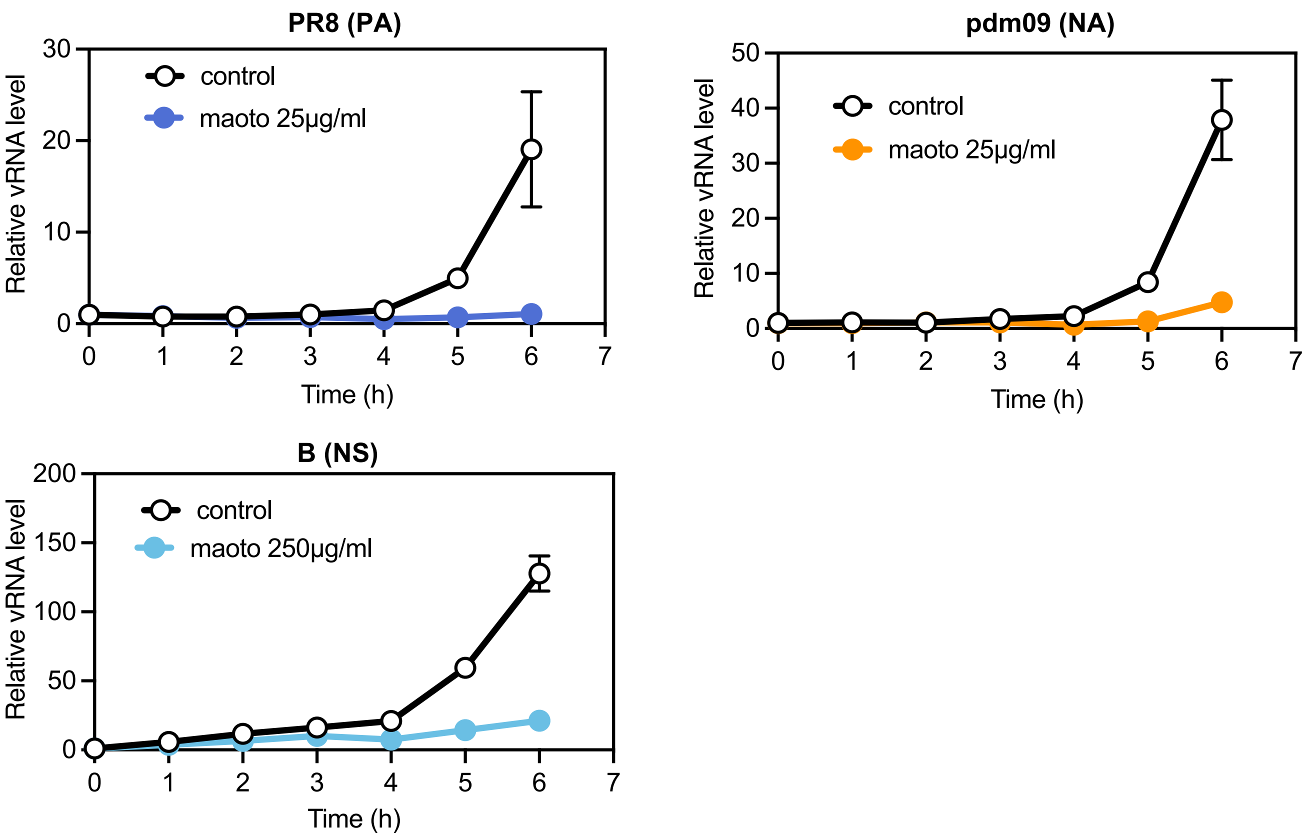

Supplementary Figure 1. Inhibitory effect of maoto during the influenza virus entry and replication phases.

Relative vRNA levels after maoto treatment (25 µg/mL for PR8 and pdm09, 250 µg/mL for B) Virus-infected cells were harvested hourly, and vRNA was quantified with qPCR using PA gene primers for PR8, NA gene primers for H1N1pdm, and NS gene primers. The data from qPCR are shown as a line graph with the 0 h time point as 1 and mean ± SD error bars (n = 3).

supplemental data fig.2

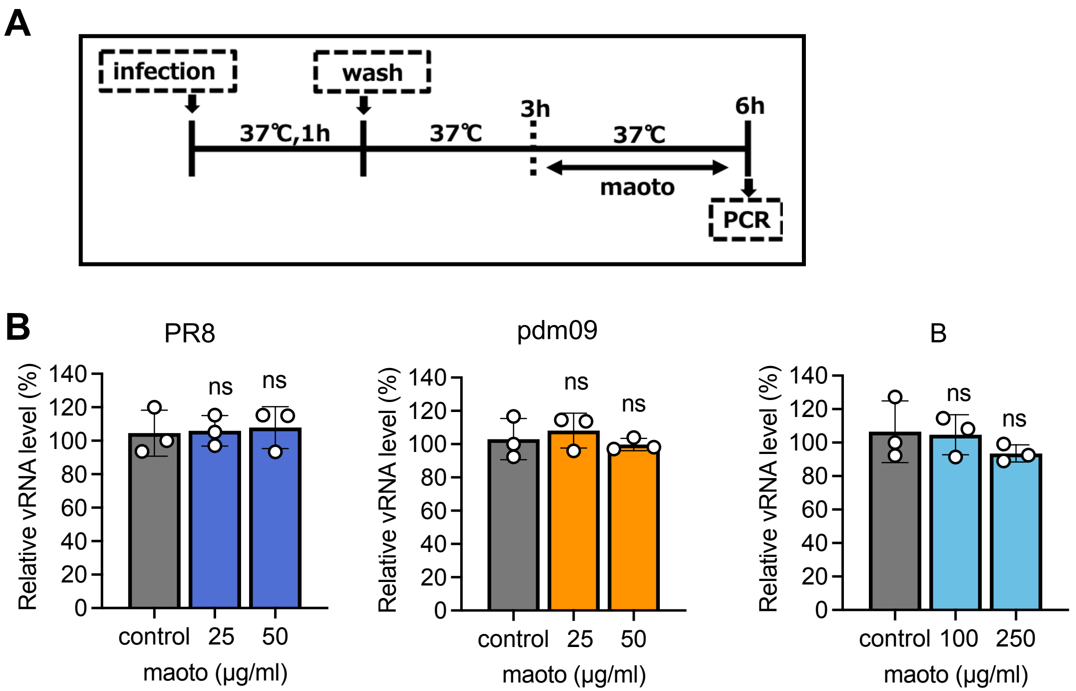

Supplementary Figure 2. Effect of maoto treatment in a late phase of viral replication.
A. Schedule for sample collection in virus infection experiments. **B.** Effect of maoto treatment 3-6 h post-infection. Cells were infected with virus for 1 h then washed with medium. After 3 h incubation, the cells were treated with the indicated concentration of maoto (25, 50 μg/mL for PR8 and pdm09, 100, 250 μg/mL for B) for 3 h, followed by vRNA measurement with qPCR. The graph shows the relative vRNA level compared to control, presented with individual values and mean ± SD error bars (n = 3). Statistical significance was determined using one-way ANOVA followed by Dunnett's posttest. ns: not significant.

supplemental data fig.3

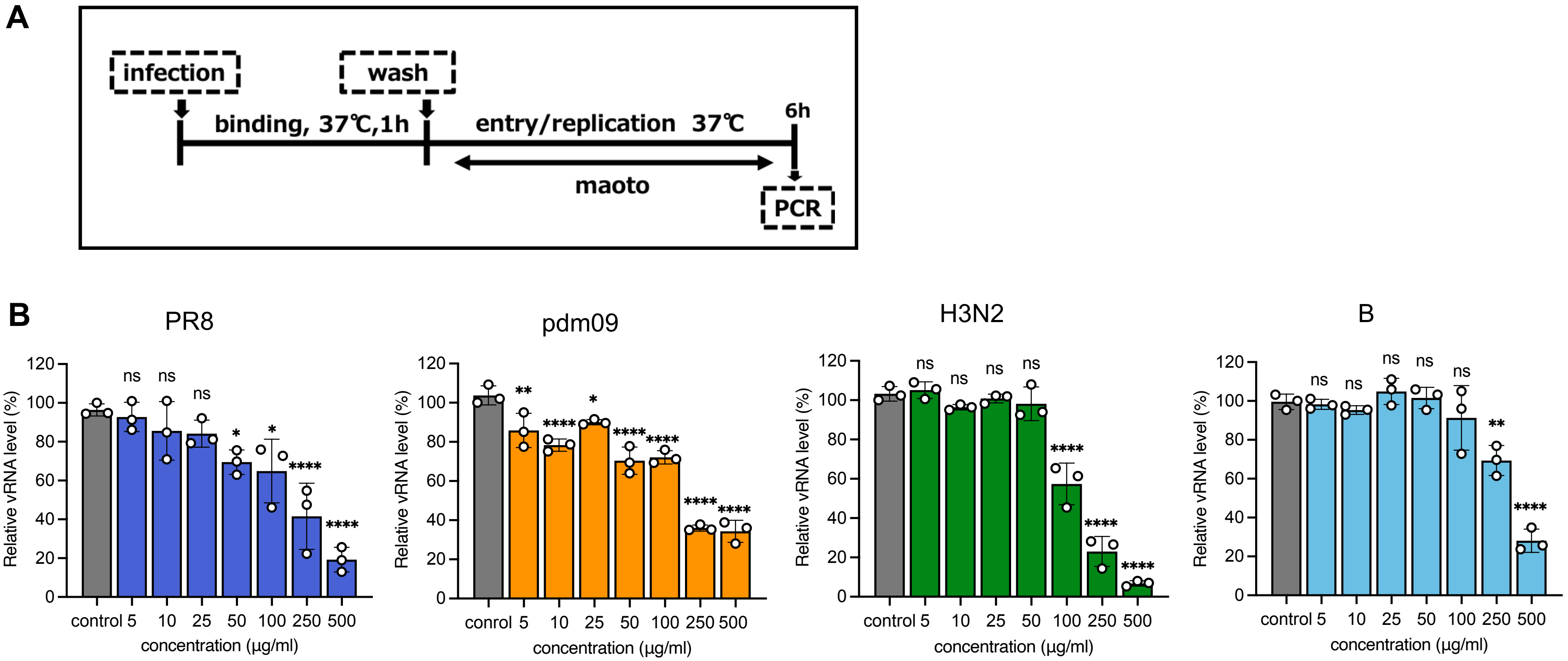

Supplementary Figure 3. Effect of maoto treatment at viral entry and in the replication phases.
A. Schedule for sample collection in virus infection experiments. **B.** Effect of maoto treatment 0-6 h post-infection. Cells were infected with virus for 1 h then washed with medium. After 6 h incubation, the cells were treated with the indicated concentration of maoto (5-500 μg/mL) followed by vRNA measurement with qPCR. The graph shows the relative vRNA level compared to control, presented with individual values and mean ± SD error bars ($n = 3$). Statistical significance was determined using one-way ANOVA followed by Dunnett's posttest. $*P < 0.05$, $**P < 0.01$, $***P < 0.001$, $****P < 0.0001$, ns: not significant.

supplemental data fig.4

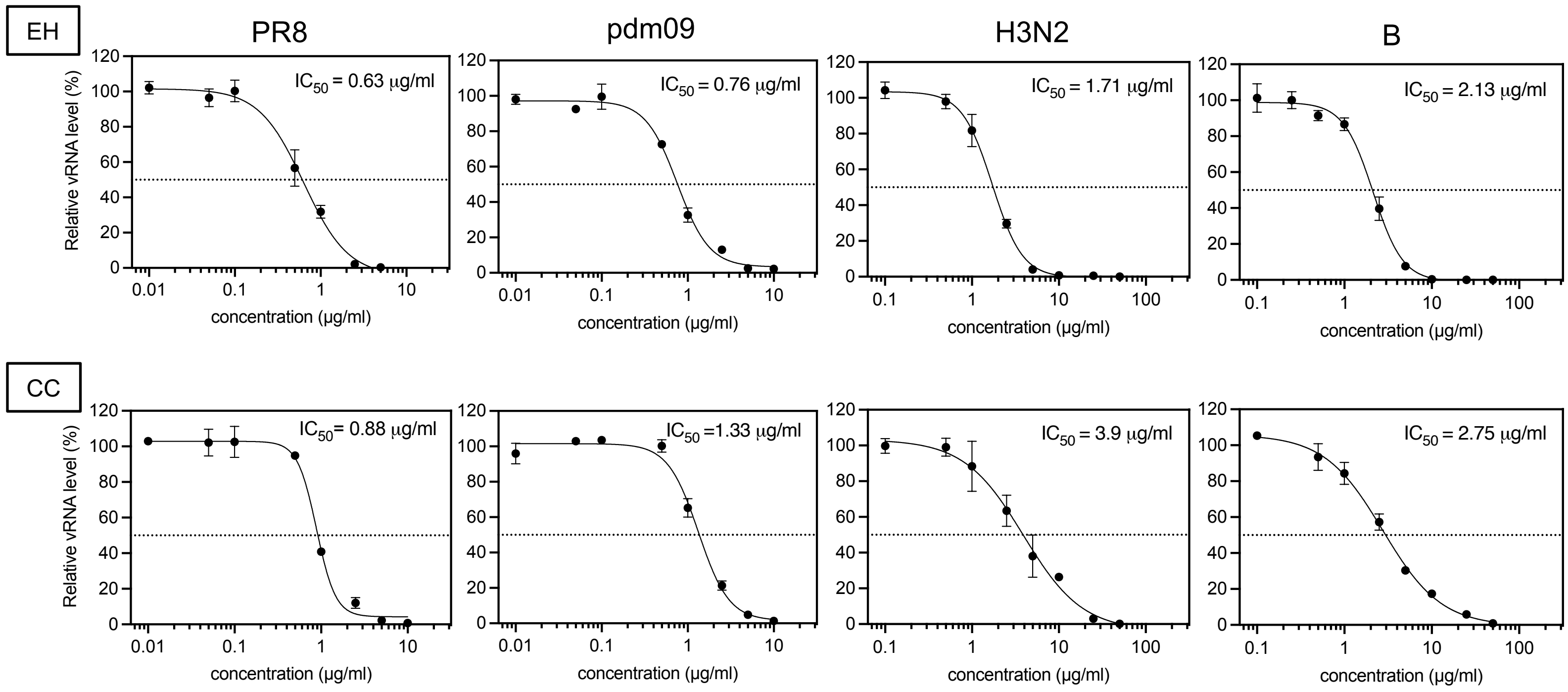

Supplementary Figure 4. IC50 Values of Anti-Influenza Virus Activity for EH and CC.
Dose-dependent decrease of vRNA by EH or CC. The viral RNA level of cells prepared after 6 h incubation was measured by RT-qPCR. IC_{50} was determined using logistic regression analysis. The graph shows relative viral RNA amounts with individual values and mean±error bars SD (n = 3).

supplemental data fig.5

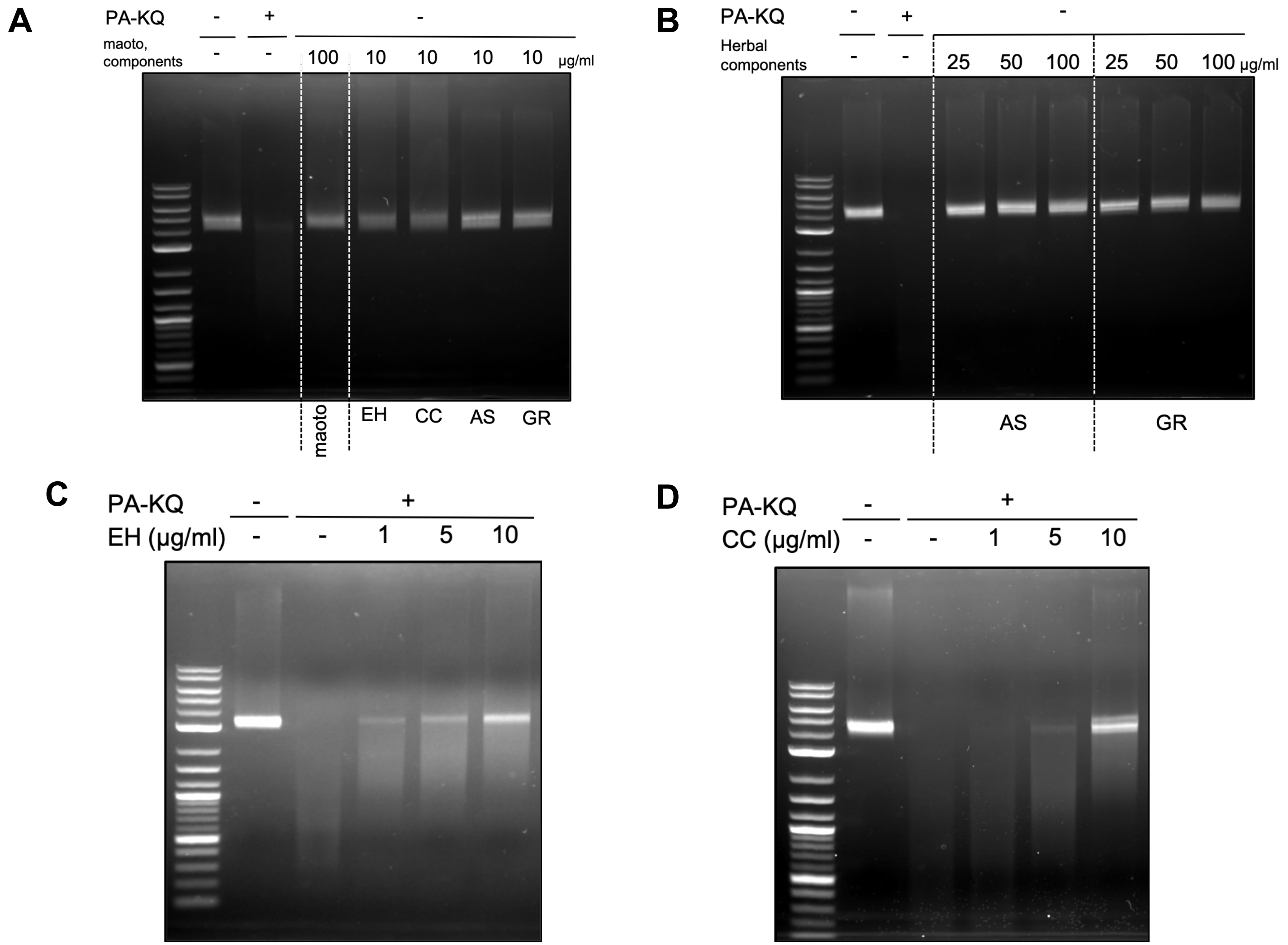

Supplementary Figure 5. Conditional setting of the influenza A virus (H1N1 PR8) PA endonuclease assay.
A. No effect of maoto or the maoto components on substrate DNA. M13mp18 ssDNA was incubated with maoto (100 μg/mL) or maoto components (10 μg/mL) in the absence of PA-KQ. The samples were analyzed by 0.8% agarose gel electrophoresis, followed by EtBr staining. **B.** No effect of AG and GR on substrate DNA. M13mp18 ssDNA was incubated with AG or GR in the absence of PA-KQ. AG and GR indicated concentrations were used. **C, D.** Effect of EH and CC on substrate DNA. M13mp18 ssDNA was incubated with EH or CC in the presence of PA-KQ. AG and GR at indicated concentrations were used.
